# Crystal structure of the membrane attack complex assembly inhibitor BGA71 from the Lyme disease agent *Borrelia bavariensis*

**DOI:** 10.1038/s41598-018-29651-9

**Published:** 2018-07-26

**Authors:** Kalvis Brangulis, Inara Akopjana, Ivars Petrovskis, Andris Kazaks, Peter Kraiczy, Kaspars Tars

**Affiliations:** 1 Latvian Biomedical Research and Study Centre, Ratsupites 1 k-1, LV-1067 Riga, Latvia; 2 Riga Stradins University, Department of Human Physiology and Biochemistry, Dzirciema 16, LV-1007 Riga, Latvia; 3 Institute of Medical Microbiology and Infection Control, University Hospital Frankfurt, Paul-Ehrlich-Str. 40, D-60596 Frankfurt am Main, Germany; 4 University of Latvia, Faculty of Biology, Jelgavas 1, LV-1004 Riga, Latvia

## Abstract

*Borrelia (B.) bavariensis*, *B. burgdorferi*, *B. afzelii*, *B. garinii*, *B*. *spielmanii*, and *B*. *mayonii* are the causative agents in Lyme disease. Lyme disease spirochetes reside in infected *Ixodes* ticks and are transferred to mammalian hosts during tick feeding. Once transmitted, spirochetes must overcome the first line of defense of the innate immune system either by binding complement regulators or by terminating the formation of the membrane attack complex (MAC). In *B*. *bavariensis*, the proteins BGA66 and BGA71 inhibit complement activation by interacting with the late complement components C7, C8, and C9, as well as with the formed MAC. In this study, we have determined the crystal structure of the potent MAC inhibitor BGA71 at 2.9 Ǻ resolution. The structure revealed a cysteine cross-linked homodimer. Based on the crystal structure of BGA71 and the structure-based sequence alignment with CspA from *B*. *burgdorferi*, we have proposed a potential binding site for C7 and C9, both of which are constituents of the formed MAC. Our results shed light on the molecular mechanism of immune evasion developed by the human pathogenic *Borrelia* species to overcome innate immunity. These results will aid in the understanding of Lyme disease pathogenesis and pave the way for the development of new strategies to prevent Lyme disease.

## Introduction

Lyme disease is the most common tick-transmitted, vector-borne disease in Europe and the US. The transmission of the etiologic agents and invasion of the mammalian host always occurs after a bite from an infected *Ixodes* tick^1–3^. Lyme disease is caused by a spirochetes belonging to the *B*. *burgdorferi* sensu lato complex that includes *B*. *burgdorferi* sensu stricto (hereafter *B*. *burgdorferi*), *B*. *afzelii*, *B*. *garinii*, *B*. *spielmanii*, *B*. *mayonii*, and *B*. *bavariensis* (previously known as *B*. *garinii* OspA serotype 4)^4–9^. Once spirochetes enter the mammalian host, they immediately face the host’s innate immune system and have to resist the host’s immune response in order to survive and disseminate to distant tissues and organs. To combat the first line immune defense *Borrelia* has developed sophisticated strategies to interfere with the complement system^10,11^.

The complement system as a part of the innate immune system is composed of numerous proteins circulating in the bloodstream as fluid phase precursors and regulators or as regulators and receptors anchored to different cell membranes^12,13^. These inactive precursors, also known as zymogens, can immediately be activated in the presence of invading pathogens^14^. The activation of the complement system is initiated by three distinct pathways: the classical, the lectin and the alternative pathways^12,13,15^. The main difference between these pathways is their initial triggers for activation. The classical pathway is primarily initiated by the binding of C1q to the antibody-antigen complex, or by binding to the surface of a pathogen while the lectin pathway is activated by the binding of mannan-binding lectin (MBL) to mannose containing carbohydrates on the microbial surface, and the alternative pathway is initiated after deposition of activated C3b molecules on the microbial surface^14,16^. Regardless of the starting point, all three pathways lead into the formation of C3 convertases that continuously cleave C3 into C3a and C3b. The deposition of C3b molecules adjacent to the C3 convertases leads to the formation of C5 convertases that cleave C5 into C5a and C5b. Binding of C5b to the surface of the pathogen initiates the sequential accumulation of components C6, C7, and C8. Once the C5b-8 complex is formed, polymerization of C9 occurs, leading to the assembly of the membrane attack complex (MAC). The assembled, slightly asymmetric, pore-like complex (as observed in electron cryo-microscopy after insertion into the cell membrane) causes a complete destruction of a wide range of Gram-negative bacteria, including spirochetes^17,18^.

Acquisition of complement regulator Factor H (CFH) is a preferred immune evasion mechanism used by a diverse number of human pathogens to mimic “self” host cells and thus protect intruders that would normally be identified as foreign particles by the complement system^15,19^. As it turns out, the pathogenic Lyme disease agents do not have a completely identical set of proteins or uniform way to interfere with the complement system^20^. *B*. *burgdorferi*, *B*. *afzelii*, and *B*. *spielmanii* resist complement-mediated killing by binding CFH and factor H-like protein 1 (CFHL-1), the key regulators in the alternative pathway^11,20–22^. As the binding of CFH is used by the host cells to inactivate the formation of C3bBb convertase, the pathogen, after binding with the complement regulators, mimics the host’s cells and is not detected as a foreign particle^19^. The borrelial outer surface proteins capable of binding CFH and CFHL-1 are designated complement regulator-acquiring surface proteins (CRASPs). In *B*. *burgdorferi*, five different CRASPs have been described so far, including CspA (also known as BBA68 or BbCRASP-1), CspZ, ErpP, ErpC and ErpA, and the crystal structures of all of these surface-exposed lipoproteins have been determined^23–27^. These proteins belong to three distinct families: CspA is a member of the PFam54 protein family; ErpA, ErpC and ErpP belong to the OspE/F-related (Erp) protein family; and CspZ does not resemble any other CRASPs of *B*. *burgdorferi*^28–30^. It has recently been shown that CspA, in addition to binding to CFH and CFHL-1, also interacts with C7, C8 and C9 (predominantly C7) and thus inhibits the formation of the MAC^31^.

Unlike serum-resistant *B*. *burgdorferi*, *B. afzelii*, and *B. spielmanii*, *B. bavariensis* does not interact with CFH and CFHL-1 or with other complement regulators^32^. Despite the lack of various CFH and CFHL-1 binding proteins, *B. bavariensis* was still found to be human serum-resistant. Two CspA orthologous proteins, BGA71 and BGA66, that are capable of binding complement system components C7, C8, and C9 and thus inhibiting the assembly of the MAC have been identified in *B. bavariensis*, allowing *B. bavariensis* to escape the attack by the immune system^32^. The binding properties of BGA66 and BGA71 showed a slight difference in their affinity for C7, C8 and C9 indicating that BGA71 predominantly interfered with C7, similar to the CspA of *B. burgdorferi*^31,32^. The importance of BGA71 and BGA66 in the resistance to the immune system was supported by the gain of resistance to complement-mediated killing after expression of either protein in the serum-sensitive *B. garinii* strain G1^32^. Apart from CspA, BGA71, and BGA66, only a few other proteins capable of inhibiting the formation of the MAC have been described. Mammalian proteins known to inhibit the formation of the MAC are located in the plasma, e.g., protectin, clusterin and vitronectin^33–35^. In addition to *B. burgdorferi*, *Trichinella spiralis* and *Schistosoma mansoni* also utilize this strategy to inhibit the assembly of the MAC^36,37^. The protein used by *T. spiralis* and *S. mansoni* is paramyosin, a structural protein that exists in different invertebrates^38^.

Considering the lack of knowledge about the molecular mechanisms of these potent MAC inhibitors, we sought to determine the crystal structure of the outer surface protein BGA71 of *B. bavariensis* to gain insights into the innate immunity resistance strategy developed by *Borrelia*. Our data reveal a fold that is similar to the crystal structure of CspA. The similarity of the fold and structure-based sequence alignment with CspA allowed us to estimate the potential binding sites for C7 and C9 that inhibit the assembly of the MAC. These findings expand our current knowledge about the pathogenesis of Lyme disease at the molecular level and might pave the way for the development of new preventive strategies that reduce the burden of Lyme disease.

## Results and Discussion

### Crystal structure of BGA71 of *B. bavariensis* PBi

**Figure 1:**
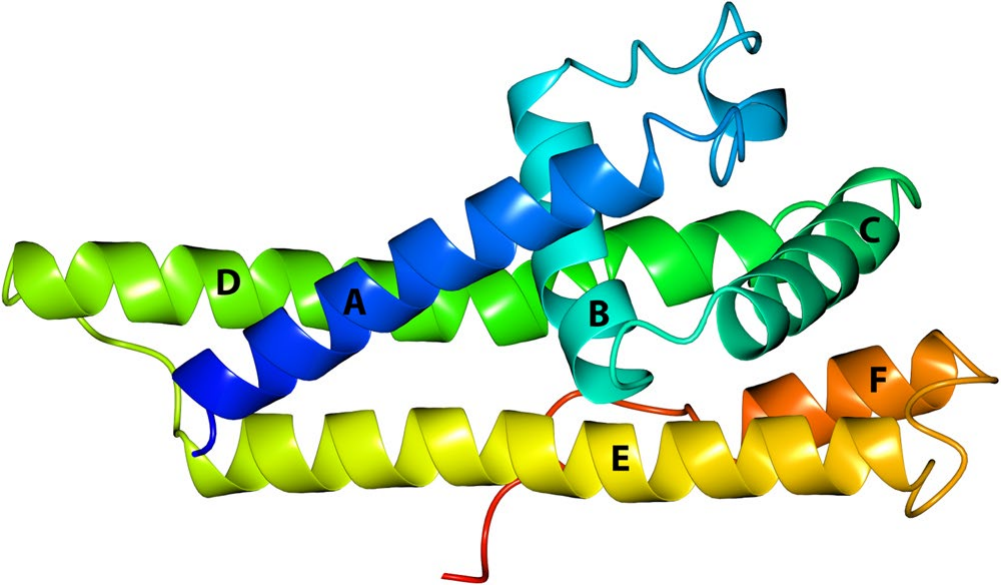
The overall structure of *B. bavariensis* BGA71 (PDB ID 6FL0). A cartoon representation of a protein monomer shown in rainbow colors from blue at the N-terminus, to red at the C-terminus. All six α-helices (A–F) are labeled.

To gain deeper insight into the molecular mechanism of complement evasion mediated by BGA66 and BGA71, we sought to crystallize the BGA71 of *B*. *bavariensis* PBi. First, the secondary structure prediction tool JPred4, in conjunction with the crystal structures of the orthologous proteins CspA, BBA64, BBA66, and BBA73 deposited in the PDB, was utilized to localize the folded protein domain and unstructured regions within BGA71^39–43^. Then, prediction was used to promote successful crystallization of BGA71 by removing the N-terminal signal sequence, as well as the unstructured N-terminal region, which is intended to connect the folded lipoprotein to the spirochetal cell surface. Thus, three different expression constructs, designated as BGA71_28-251_, BGA71_49-251_ and BGA71_62-251_, were generated. While the BGA71_28-251_ construct excluded the predicted signal sequence region, BGA71_49-251_ and BGA71_62-251_ also lacked the N-terminal unstructured region. All three BGA71 constructs were overexpressed in *E. coli*, but only BGA71_62-251_ yielded crystals, suggesting that the flexible region present in the other two constructs interfered with the crystal formation, as previously observed for the BBA66 protein of *B. burgdorferi*^39^. The diffracting crystals of the Se-Met BGA71_62-251_ construct belonged to the space group P3_1_ and contained 18 molecules per asymmetric unit with a water content of approximately 45%. The crystals used for collection of native diffraction data also belonged to the space group P3_1_, but the unit cell size was smaller and only six molecules were found per asymmetric unit with a water content of approximately 45%. *B. bavariensis* BGA71 is an α-helical protein composed of six α-helices (designated A-F) connected by several loops **(**Fig. 1**)**.

The final model of BGA71_62-251_ was generated for residues 69–251 since the first four residues that remained after cleavage of the His-tag and residues 66–68 were not built due to weak electron density. The overall fold of the BGA71 protein looks similar (C^α^ root-mean-square deviation from 1.66 Ǻ to 2.63 Ǻ) to the previously released crystal structures of the orthologous PFam54 proteins CspA, BBA64, BBA66, and BBA73 of *B. burgdorferi*^39–42^. In addition to the already mentioned CspA protein, which is essential for *B. burgdorferi* to escape the innate immunity, the other PFam54 family members (BBA70, BBA64, BBA66, and BBA73) are also known to play distinct roles in the pathogenesis of Lyme disease^31,44–47^. BBA64, for example, is essential to ensure the transfer of bacteria from the tick to the host during the tick’s blood meal, while BBA66 is an important infection-associated protein involved in the transmission and subsequent dissemination of *B. burgdorferi* from infected *Ixodes* ticks to the mammalian host^44,45^. Previously, BBA70 was identified as a plasminogen-binding protein enabling spirochetes to migrate away from the port of entry and enter other tissues by degrading host physiological barriers, such as the extracellular matrix or the basement membranes^48,49^. Furthermore, by investigating DALI and PDBeFold analysis (three-dimensional alignment with other protein structures), we did not find any structural similarities between BGA71 and other bacterial proteins, except the members of the *B. burgdorferi* PFam54 proteins^50,51^. These results suggest that the fold used by BGA71 and the orthologous protein CspA to bind to the complement components C7, C8 and C9 is unique and has never been described in any other terminal pathway inhibitors of the complement system.

### Dimerization of BGA71 by cysteine cross-linking

**Figure 2:**
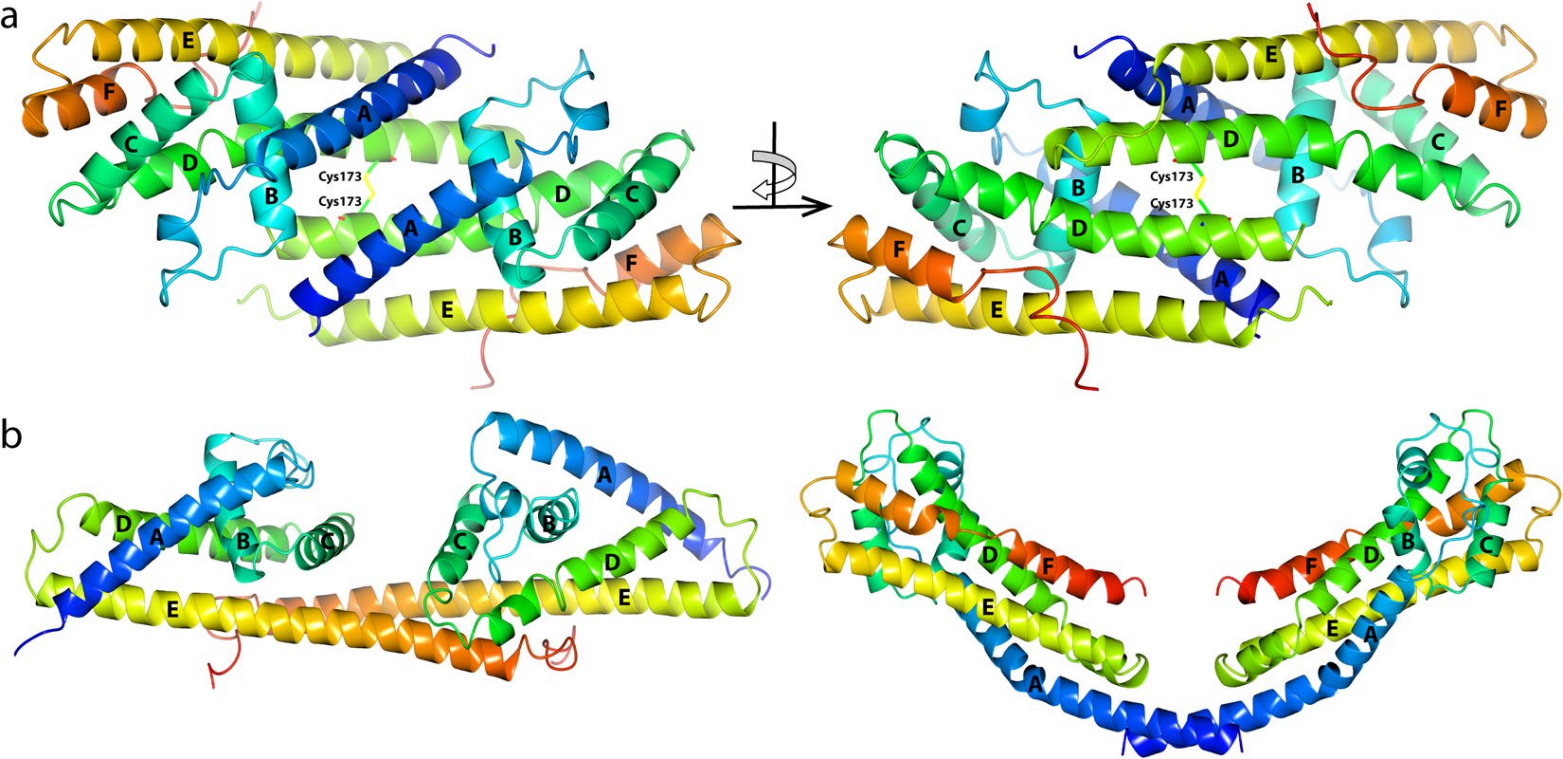
Dimerization of BGA71 and orthologous proteins CspA and BBA73. (**a**) Cysteine cross-linked BGA71 homodimer as observed in the crystal structure (PDB ID 6FL0). Cartoon representation of BGA71 homodimer at two different angles that differ by 180 degrees. (**b**) Homodimer of PFam54 family members CspA (left) and BBA73 (right). The color is used to indicate the start and end of the protein monomer, running from blue at the N-terminus to red at the C-terminus. α-helices are labeled, starting from the N-terminus of the protein.

**Figure 3:**
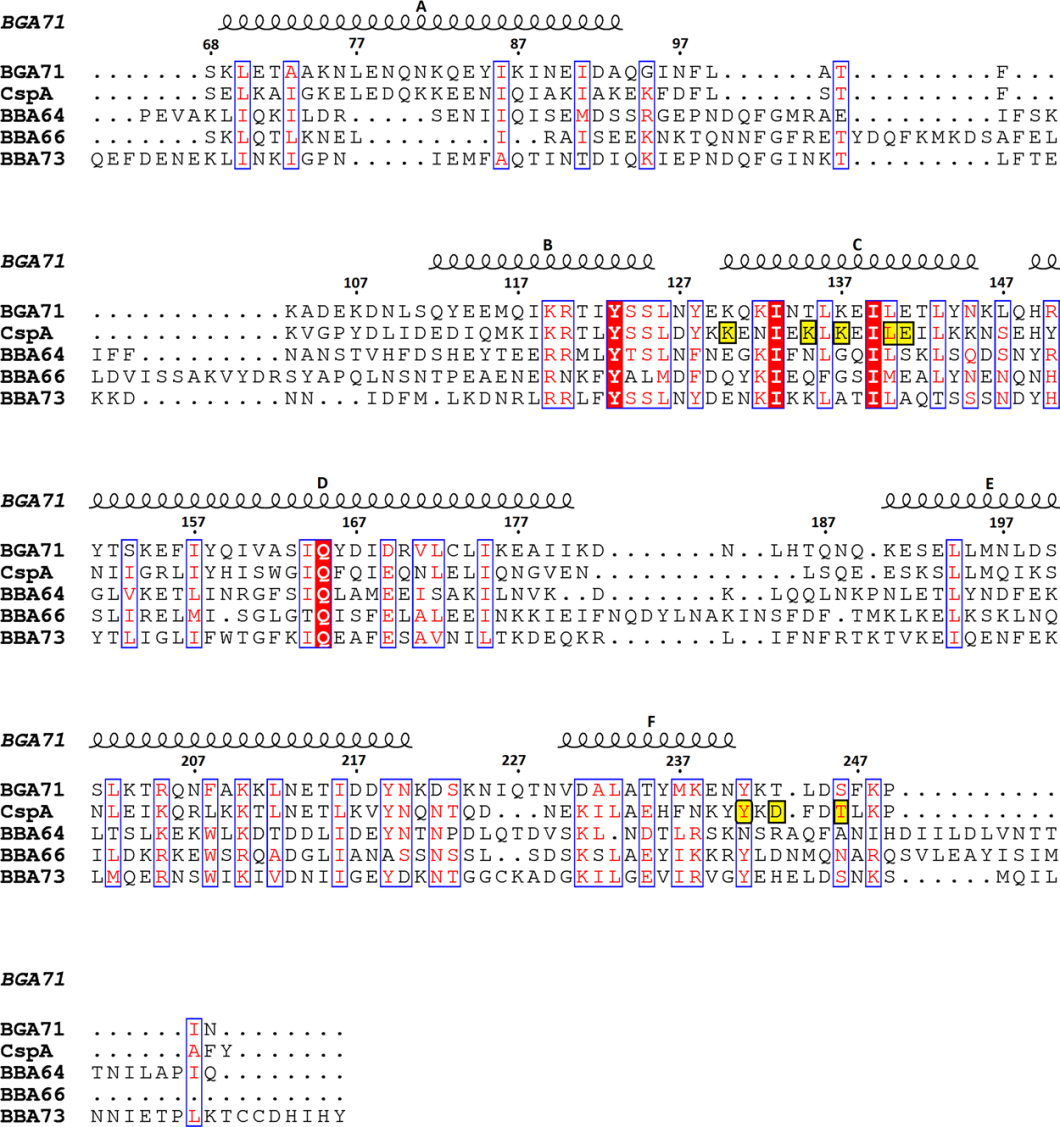
Sequence alignment of BGA71 and PFam54 family members CspA, BBA64, BBA66 and BBA73. Conserved residues are indicated in red and framed. Secondary structure elements are illustrated above the alignment for BGA71. Only the residues found in the crystal structures of the respective proteins have been used in the alignment, thus excluding the unstructured and signal sequence containing N-terminal part. Numbering above the sequence is shown for BGA71. Residues encompassing region 1 and region 3 in CspA used for the mutational analysis are framed in yellow background. The sequence alignment was generated by ESPript 3^67^.

By surveying the crystal structure of BGA71, we identified cysteine 173 in α-helix D as the residue that forms disulfide bridges, which is responsible for cross-linking of the two monomers (Fig. 2a). This finding was also confirmed by employing a gel-filtration analysis. Dimerization has also been observed for PFam54 family members CspA and BBA73^27,41^. The structural prerequisites causing dimerization and the formed dimer interface between BGA71 and both orthologous proteins are different and the interactions involved in CspA and BBA73 dimerization are non-covalent, opposite to BGA71 (Fig. 2b). Furthermore, comparative sequence analysis of the PFam54 orthologous proteins reveals that cysteine 173 is not present in any other orthologous PFam54 protein, suggesting that dimerization by cysteine cross-linking is a unique feature of BGA71 (Fig. 3). Thus, it is tempting to speculate that these cysteine-directed disulfide bridges are of biological relevance and expose a highly stable homodimer on the outer surface of *B. bavariensis*.

### Structural comparison of BGA71 with the PFam54 protein family members CspA, BBA64, BBA66, and BBA73

**Figure 4:**
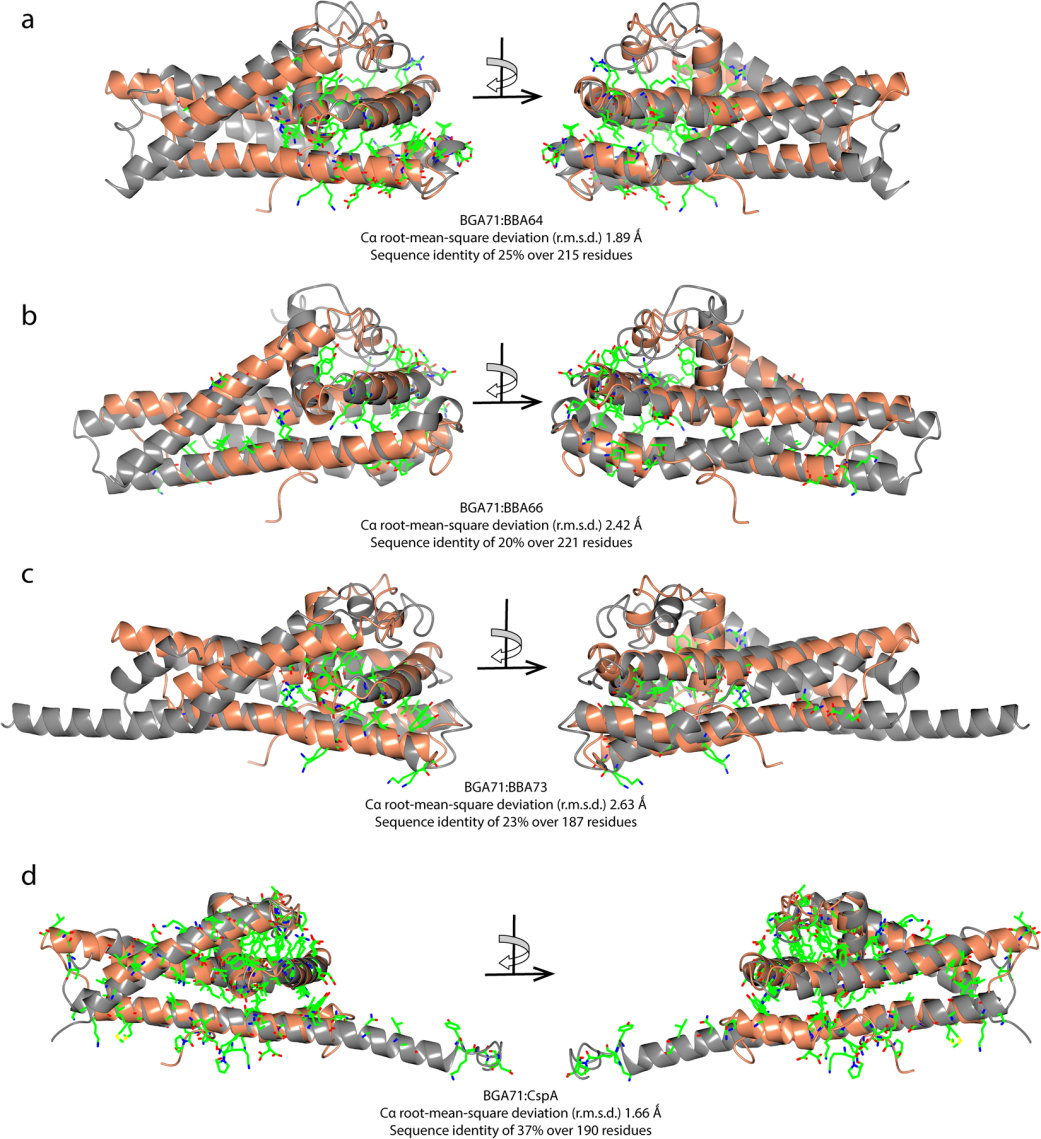
Superimposed crystal structures of BGA71 and four orthologous proteins in the PFam54 family of *B. burgdorferi*. (**a**) BGA71 and BBA64 (PDB ID 6FL0 and 4ALY), (**b**) BGA71 and BBA66 (PDB ID 6FL0 and 2YN7), (**c**) BGA71 and BBA73 (PDB ID 6FL0 and 4AXZ), (**d**) BGA71 and CspA (PDB ID 6FL0 and 5A2U). Superimposed crystal structures are illustrated at two different angles differing by 180 degrees. BGA71 is shown in orange, while the corresponding superimposed crystal structure is in gray. The conserved side chains are shown in both superimposed molecules. Data for the superimposed structure C^α^ root-mean-square deviation (r.m.s.d.) and primary sequence identity are positioned below the alignment.

To estimate the conservation of the overall folds and to identify relevant conserved residues, a sequence alignment of BGA71 and PFam54 family members was conducted in addition to structure-based comparison using the atomic structure of BGA71 and the structures of the orthologous proteins CspA, BBA64, BBA66, and BBA73 (Figs 3 and 4). The comparative analysis revealed that the overall folds of BGA71 and BBA64 are very similar (C^α^ root-mean-square deviation 1.89 Ǻ), exhibiting minor differences in the loop regions but major differences at the C-terminus (Fig. 4a). The terminal α-helix F in BGA71 is much shorter and switches to a loop region, while the terminal α-helix in BBA64 is extended, covering the side of the protein. The conserved residues in both proteins are mainly found within α-helices B, C and E in a very compact formation dominated by hydrophobic interactions and ionic bonds, apparently to sustain the proper fold of the proteins.

Similar conclusions can be made from the superimposed crystal structures of BGA71 and BBA66 (Fig. 4b). The C-terminal α-helix in BBA66 is extended in the same way as observed in BBA64 and the conserved residues residing mainly in helices C, E and F are suggested to be involved in the stabilization and maintenance of the overall protein fold due to their hydrophobic character or their location, which is towards the core region.

As for BBA73, the crystal structure is more distinct from BGA71 than the other PFam54 members, with the conserved residues located within a small core region encircling α-helices B, C, and E (Fig. 4c). Moreover, the superimposed crystal structures revealed significant differences not only at the C-terminal α-helix, as observed for BBA64 and BBA66 but also in the N-terminal α-helix A. As shown in Fig. 4c, the N-terminal α-helix A in BBA73 is 20 amino acids longer than the α-helix A of BGA71, and the extended α-helix in BBA73 is important to ensure dimerization^41^.

Overall, BBA64, BBA66 and BBA73 play different roles in the pathogenesis of Lyme disease, as described previously, and these proteins indicate a low identity with BGA71^32,44,45,47^. Additionally, a few conserved residues between the proteins, as judged from the sequence alignment and superimposed crystal structures, appear to be required for proper folding and maintenance of the common fold (Figs 3 and 4). This is due to their hydrophobic nature and location towards the protein core region and formation of internal salt bridges between conserved polar residues. Therefore, we assume that there is no functional similarity between BGA71 and the PFam54 family proteins BBA64, BBA66 and BBA73, except for their common fold.

**Figure 5:**
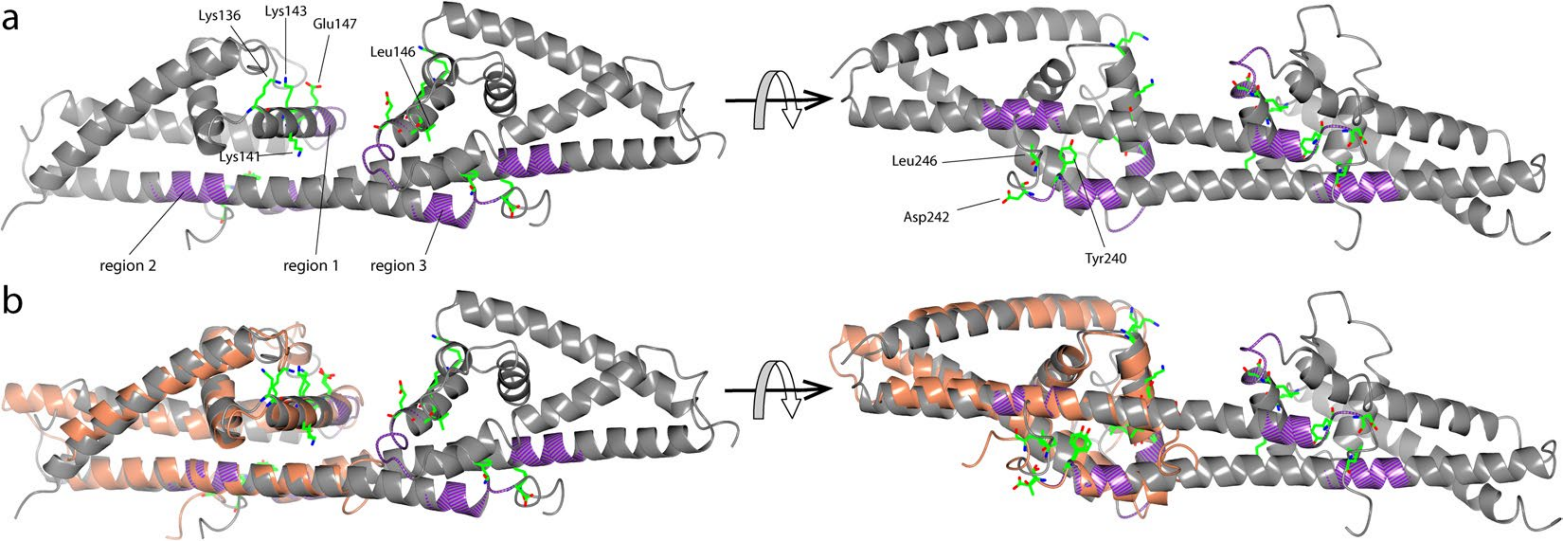
Conserved residues of BGA71 and CspA (PDB ID 6FL0 and 5A2U). (**a**) Three regions, region 1 (residues 146-LEILKKNSE-154), region 2 (residues 204-LEIKQRLKKT-213), and region 3 (residues 233-AEHFNKYYKD-242), thought to be involved in the binding of CFH and CFHL-1 are illustrated in CspA as segments (purple). Key side chains of residues involved in the binding of CFH and CFHL-1 are illustrated as bonds. (**b**) Superposition of CspA and BGA71 crystal structures with the key residues illustrated in CspA and the respective residues in BGA71. BGA71 is shown in orange while CspA is in gray. CspA is shown as a homodimer.

As depicted in Fig. 4d, the structure of CspA showed the highest similarity to BGA71 among the other PFam54 orthologs of *B*. *burgdorferi*, with a C^α^ root-mean-square deviation of 1.66 Ǻ. CspA is known to interact with complement regulators CFH and CFHL-1, as well as with complement components C7 and C9^29,31^. While BGA71 did not bind CFH and CFHL-1, an interaction with components C7 and C9 has been demonstrated^32^. Thus, a structure-based sequence alignment was analyzed to explain the distinct binding patterns of CFH and CFHL-1 and to decipher the conserved residues potentially involved in their interactions with C7 and C9 (Fig. 3). In an initial attempt, based on a factor-H binding motif search and pepspot analysis, three regions in the CspA molecule have been proposed to be involved in the binding of CFH and CFHL-1^29^ (Fig. 5). Later, a homodimer was proposed as the functional unit for the binding of CFH and CFHL-1^42^. Additionally, by employing *in vitro* mutagenesis, the binding of CFH and CFHL-1 in CspA was attributed to several key residues, confirming the assumption that homodimer formation in CspA is essential for the binding of CFH and CFHL-1^52^ (Figs 3 and 5a). By investigating site-directed mutagenesis of residues Tyr240, Asp242 and Leu246 (corresponding to Tyr241, Thr243 and Leu247 in BGA71) in region 3, binding of CFH and CFHL-1 to CspA was completely abolished by disrupting the homodimer and, more importantly, the binding pocket at the dimer interface^52^. While BGA71 cannot form a homodimer in the same manner as CspA due to the structural differences in the C-terminal α-helix F, the residues close to the C-terminus are well conserved between BGA71 and CspA (Fig. 3). By superposition of the crystal structures, we see that the conserved residues are important to place in the exact location the C-terminal α-helix between α-helices D and E (Fig. 4d). In the CspA homodimer, the C-terminal α-helix E is nestled along the C-terminal α-helices D and E of the other monomer, while in BGA71 the C-terminal α-helix E makes a turn and runs backward in exactly the same position as observed in the dimer interface of CspA. In addition, the last 10 C-terminal residues that form the unstructured loop region are positioned very similarly in both the BGA71 and CspA homodimers.

Concerning region 1, which contains the α-helix C that is located at the dimer interface in CspA, mutations of residues Lys136, Lys141, Lys143, Leu146 and Glu147 (corresponds to Lys130, Thr135, Lys137, Leu140 and Glu141 in BGA71) significantly reduced the binding of CFH and CFHL-1^42,52^. This region encompassing the abovementioned residues, which has been found to be important for the binding of both complement regulators, is well conserved in both borrelial proteins, suggesting that BGA71 should be able to bind CFH and CFHL-1 similar to CspA (Figs 3 and 5b). However, the lack of binding complement regulators can be explained by the inability of BGA71 to form a homodimer in the same manner as CspA. This means that it cannot form a binding pocket that has the same dimensions found in the dimer cleft of CspA^53^. The difference in the manner of dimerization was confirmed only from the crystal structure of BGA71 since previously it was thought that the C-terminal region of BGA71 was similar to that of CspA^32^. While the cleft formed at the CspA homodimer is the most likely binding site for CFH and CFHL-1, it is equally likely that the binding of complement regulators by CspA is not limited to the abovementioned residues and that some other relevant residues, which have not been selected in the previous mutagenesis analysis, are involved and are not conserved between CspA and BGA71.

### Potential binding site in BGA71 for the complement components C7 and C9

**Figure 6:**
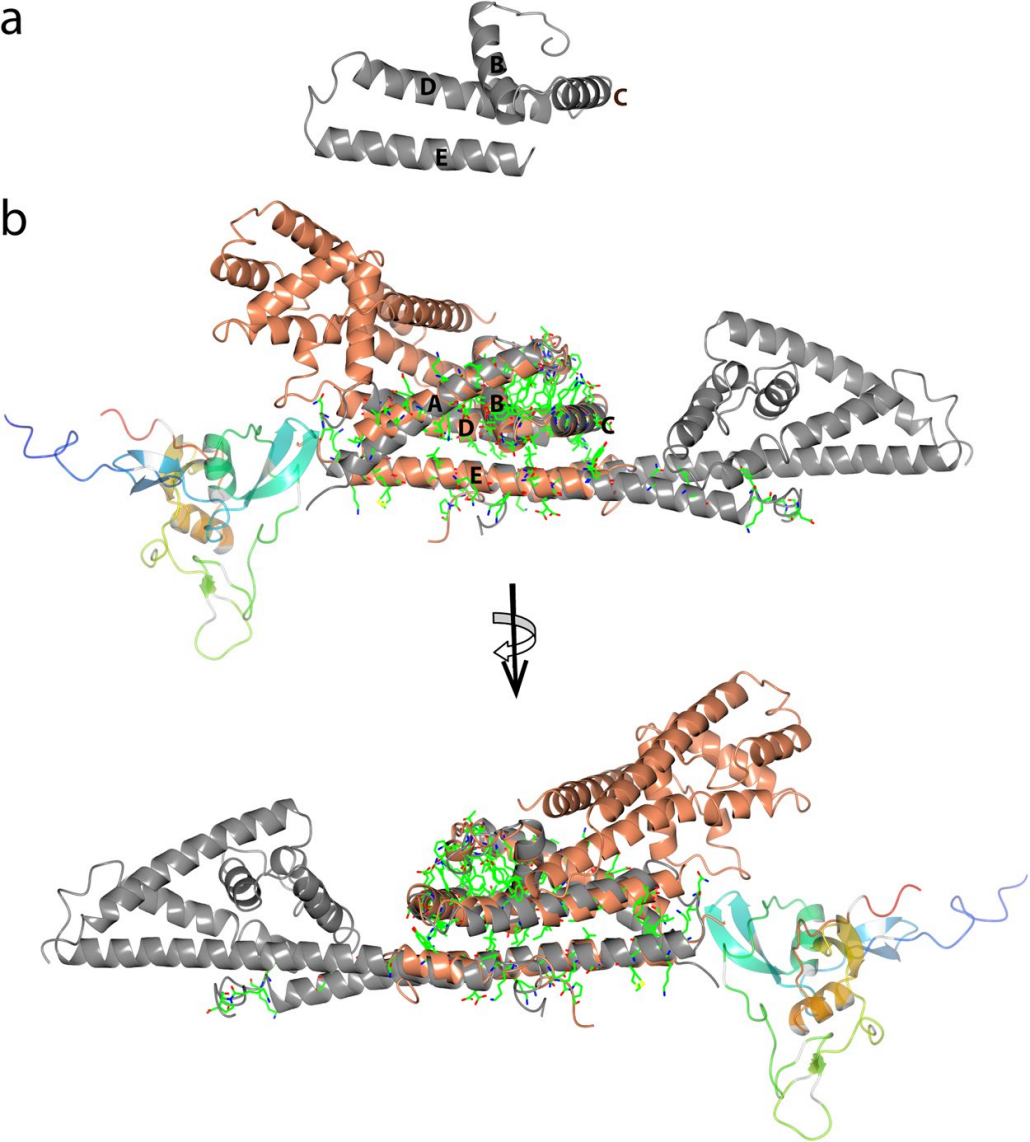
Potential interaction site for complement component C7 for BGA71 and CspA. (**a**) The part of the CspA structure (residues 109-215) which has been predicted to interact with the complement proteins C7 and C9. (**b**) Superposition of BGA71 and CspA crystal structures (PDB ID 6FL0 and 5A2U) with the conserved residues illustrated as bonds. BGA71 and CspA are illustrated as homodimers. Crystal structure of complement protein C7 (PDB ID 2WCY) is illustrated in rainbow colors from blue at the N-terminus to red at the C-terminus. BGA71 is shown in orange, while CspA is in gray.

It has been shown that the binding site for C7 and C9 is distinct from the site involved in the binding of CFH and CFHL-1 since C7 and C9, as well as both complement regulators, can bind simultaneously to CspA^31^. Several N-terminal and C-terminal truncation mutants of CspA did not influenced the binding of C7 and C9, suggesting that the C-terminus and the formed homodimer are not essential for the binding of both complement components, as was demonstrated for CFH and CFHL-1^31,53^. Overall based on the results obtained from CspA mutagenesis studies the major C7 and C9 binding region was proposed to reside within residues 109-215^31^ (Fig. 6a).

Previously, functional analyses using a chimeric protein consisting of the N-terminal part of ZQA71, an orthologous protein that belongs to the PFam54 family from *B. garinii* ZQ1, lacking complement-inhibitory activity, and the C-terminal part of BGA71 revealed that this molecule is unable to inhibit the complement system suggesting that the binding site for C7 and C9 resides at the N-terminus^32^. However, the proposed N-terminal residues 37–63 involved in the binding of C7 and C9 are not visible in the crystal structure of BGA71 since they form a flexible loop region connecting the protein structural domain to the cell surface. Considering the abovementioned conditions regarding the binding of C7 and C9, and assuming that the interaction occurs in a similar manner between BGA71 and CspA, we propose that the potential binding site for C7 and C9 is located in α-helices A and E. In favor of this assumption is the structural prediction of protein-protein interactions by using pyDock software and the crystal structure of complement protein C7 factor-I-like module (PDB ID 2WCY) which indicated that the most likely C7/C9-interaction site should be located in α-helices A and E in both BGA71 and CspA^54,55^ (Fig. 6b). It is noteworthy that there is no crystal structure of the full-length C7 available. Thus, we cannot exclude the possibility that C7 is associated with regions other than the factor-I-like module of C7.

Nevertheless, based on structural data and previous data analysis, locating the C7/C9-interaction site in other regions is very unlikely because all C-terminal deletion mutants of CspA that were analyzed so far were able to bind C7 and C9, excluding the necessity for the C-terminal end of α-helix E in CspA or α-helix F in BGA71^31^. Meanwhile, the region in CspA around α-helix C is involved in binding of CFH and CFHL-1, but the binding with components C7 and C9 is found to be simultaneous^31^. In addition, α-helices A, B and D of CspA could also potentially serve as binding sites for C7 and C9, but in BGA71, the same region is not exposed and accessible because of the formed homodimer interface. In addition, our proposed binding site in BGA71 for complement components C7 and C9 is close to the N-terminus, which is in agreement with the previously obtained results that the N-terminus is involved in interaction with C7 and C9^32^.

In conclusion, we have solved the crystal structure of the key complement inhibitory protein BGA71 from *B. bavariensis*. Investigating structural comparisons with the orthologous protein CspA of *B. burgdorferi* known to be capable of binding C7 and C9, the potential binding site for these two complement components has been proposed. Our findings contribute to the understanding of the pathogenesis of Lyme disease as they reveal the molecular mechanisms allowing *B. bavariensis* to overcome the host’s innate immunity.

## Materials and Methods

### Cloning and expression of native and selenomethionine labeled BGA71

The *Bga71* gene encoding the outer surface protein BGA71 was PCR-amplified from *B. bavariensis* PBi genomic DNA. Three different PCR-amplified products were made, designated BGA71_28-251_, BGA71_49-251_ and BGA71_62-251_, by excluding the unstructured N-terminal region and the predicted signal sequence (oligonucleotides used for PCR are listed in Table 1). The amplified PCR products were designated for ligation into the NcoI and NotI restriction sites of the expression vector pETm-11. Expression vectors harboring the respective borrelial genes were transformed into *Escherichia coli* RR1 cells and positive clones were selected on LB agar plates containing Kanamycin (10 mg/ml) at 37 °C for 24 h. Colonies selected were transferred into a 2 mL Kanamycin-supplemented LB medium, incubated at 37 °C for 24 h and harvested by centrifuging at 5000 g for 10 min. Plasmids were isolated, and the inserted DNA fragments were verified by sequencing. Only plasmids with the correct sequences were used for transformation of *E. coli* BL21 (DE3) cells. The resulting transformants were cultivated in 2xTYP media (supplemented with kanamycin, 133 mM phosphate buffer (pH 7.4) and glucose (4 g/l)) with vigorous agitation at 25 °C until an OD_600_ of 0.8–1.0 was achieved. Protein expression was induced by the addition of 0.2 mM isopropyl thio-β-D-galactoside and cells were cultivated for 16 h at 25 °C. The expression of L-selenomethionine (Se-Met)-labeled protein was carried out as described previously^56^.

**Table 1:** Oligonucleotides used in this study.

Oligonucleotide	Sequence (5′-3′)	Application
BGA71 F1	CATGCCATGGGCGATAAAATAGACCTGGAA	Used for amplification of BGA71_28-251_
BGA71 F2	CATGCCATGGGCAATCAAAAAACTAAAGCC	Used for amplification of BGA71_49-251_
BGA71 F3	CATGCCATGGGCAATCAAAAAACTAAAGCC	Used for amplification of BGA71_62-251_
BGA71 R	GCTTGCGGCCGCTTAATTAATAGGCTTAAAAGA	Used for amplification of BGA71

### Purification and His-tag cleavage of BGA71

To purify the His-tagged protein, *E. coli* cells were resuspended in a lysis buffer (25 mM NaH_2_PO_4_, 50 mM NaCl, 10 mM imidazole) and disrupted by sonication. Cell debris was removed by centrifuging at 10 000 g for 30 min at 4 °C. The supernatant was then transferred to a Ni-NTA agarose (Qiagen, Germany) column and the bound His-tagged protein was eluted from the column with an elution buffer (300 mM imidazole (pH 7.5), 250 mM NaCl). To remove imidazole prior to the crystallization trials, the buffer was exchanged by 10 mM Tris-HCl (pH 8.0) using an Amicon centrifugal filter unit (Millipore, Germany).

The N-terminal His-tag was removed by adding TEV protease and the reaction mixture was incubated for 16–20 h at room temperature. Both the cleaved His-tag and TEV protease were removed by loading the reaction mixture onto a Ni-NTA column (Qiagen, Germany), and the flow through containing the cleaved protein was concentrated to 13 mg/ml using an Amicon centrifugal filter unit (Millipore, Germany).

### Estimation of the multimeric state by gel filtration chromatography

Protein samples with concentrations of 3–5 mg/ml in 10 mM Tris-HCl (pH 8.0) containing 250 mM or 500 mM NaCl were loaded onto a pre-packed Superdex 200 10/300 GL column (Amersham Biosciences, UK). The column was pre-equilibrated with the same buffer and the applied protein sample was run at a flow rate of 0.7 ml/min. Bovine serum albumin (67 kDa), ovalbumin (43 kDa) and chymotrypsinogen A (25 kDa) were used as protein molecular weight standards.

### Crystallization of BGA71

The crystallization of BGA71 was set by the Tecan Freedom EVO100 workstation (Tecan Group Ltd., Switzerland) in sitting-drop 96-well plates by mixing 0.4 μl of 13 mg/ml protein with 0.4 μl of precipitant solution from JCSG-*plus* sparse matrix screen (Molecular Dimensions Ltd., UK). The most promising conditions for BGA71 were optimized and rectangular crystals in conditions containing 26% PEG 3350, 0.1 M Tris-HCl (pH 6.0) and 0.2 M calcium chloride were obtained. Crystallization conditions for the Se-Met derived BGA71 protein were the same as for the native protein. Before data collection, the crystals were harvested by using 25% ethylene glycol as a cryoprotectant and stored in liquid nitrogen.

### Data collection, structure determination, and refinement

The X-ray diffraction data measurements were carried out at the MX beamline instrument BL 14.1 at Helmholtz-Zentrum, Berlin. The structure of BGA71 was determined by using the single-wavelength anomalous dispersion (SAD) method, and the native crystal structure of BGA71 was determined by molecular replacement using the Se-Met derived structure of BGA71 as a model (PDB ID 6FL0). The crystal structure of CspA was determined by molecular replacement using the previously determined structure as a search model (PDB ID 1W33)^27^. The reflections were indexed by MOSFLM and XDS and scaled by SCALA and AIMLESS from the CCP4 suite^57–59^. For determination of Se-Met BGA71, the phases were obtained by using SHELX C/D/E^60^. For molecular replacement, the phases were determined by Phaser^61^. The initial protein model was built automatically in BUCCANEER^62^, and minor re-building of the model was performed manually in COOT^63^. The solvent content in the unit cell was determined by MATTHEWS^64^. Crystallographic refinement was performed with REFMAC5^65,66^. A summary of the data collection, refinement and validation statistics for native and Se-Met derived BGA71 is given in Table 2.

**Table 2:** Statistics for Data and Structure Quality.

**Dataset**	**SeMet**	**Native**
**Space group**	P 3_1_	P 3_1_
**Unit cell dimensions**		
a (Å)	239.20	137.06
b (Å)	239.20	137.06
c (Å)	56.25	56.41
Wavelength (Å)	0.9796	1.0000
Resolution (Å)	119.59-2.90	68.54-2.80
Highest resolution bin (Å)	2.96-2.90	2.87-2.80
No. of reflections	833897	82244
No. of unique reflections	79678	28251
Completeness (%)	99.9 (100.0)	96.9 (94.2)
R_merge_	0.08 (0.23)	0.11 (0.32)
*I/σ* (*I*)	19.9 (9.7)	7.3 (2.7)
Multiplicity	10.5 (10.5)	2.9 (2.7)
**Refinement**		
R_work_	0.192 (0.195)	0.189 (0.144)
R_free_	0.248 (0.282)	0.216 (0.181)
Average B-factor (Å^2^)		
Overall	32.1	39.9
From Wilson plot	15.4	8.1
**No. of atoms**		
Protein	53056	16956
Water	0	0
**RMS deviations from ideal**		
Bond lengths (Å)	0.006	0.039
Bond angles (^o^)	1.065	1.970
**Ramachandran outliers (%)**		
Residues in most favored regions (%)	97.30	97.99
Residues in allowed regions (%)	2.70	2.01
Outliers (%)	0.00	0.00

*Values in parentheses are for the highest resolution bin.

### Accession codes

The final coordinates and structure factors for Se-Met and native *B. bavariensis* BGA71 have been deposited in the Protein Data Bank with the accession numbers 6FL0 and 6FMH.
